# Clinicians adopting evidence based guidelines: a case study with thromboprophylaxis

**DOI:** 10.1186/1472-6963-11-240

**Published:** 2011-09-28

**Authors:** Nicola H Chapman, Steven P Lazar, Margaret Fry, Marissa N Lassere, Beng H Chong

**Affiliations:** 1 St George Public Hospital, Kogarah, NSW, Australia; 2 St George Clinical School, University of New South Wales, Sydney, Australia; 3 Faculties of Nursing and Midwifery, University of Technology, Sydney, Australia

## Abstract

**Background:**

Venous Thromboembolism (VTE) is a cause of hospital mortality and managing its morbidity is associated with significant expenditure. Uptake of evidenced based guideline recommendations intended to prevent VTE in hospital settings is sub-optimal. This study was conducted to explore clinicians' attitudes and the clinical environment in which they work to understand their reluctance to adopt VTE prophylaxis guidelines.

**Methods:**

Between February and November 2009, 40 hospital employed doctors from 2 Australian metropolitan hospitals were interviewed in depth. Qualitative data were analysed according to thematic methodology.

**Results:**

Analysis of interviews revealed that barriers to evidence based practice include i) the fragmented system of care delivery where multiple members of teams and multiple teams are responsible for each patient's care, and in the case of VTE, where everyone shares responsibility and no-one in particular is responsible; ii) the culture of practice where team practice is tailored to that of the team head, and where medicine is considered an 'art' in which guidelines should be adapted to each patient rather than applied universally. Interviewees recommend clear allocation of responsibility and reminders to counteract VTE risk assessment being overlooked.

**Conclusions:**

Senior clinicians are the key enablers for practice change. They will need to be convinced that guideline compliance adds value to their patient care. Then with the support of systems in the organisation designed to minimize the effects of care fragmentation, they will drive practice changes in their teams. We believe that evidence based practice is only possible with a coordinated program that addresses individual, cultural and organisational constraints.

## Background

Health systems around the world are under financial pressure and reform measures are intensifying. Such measures often emerge as efforts to standardise clinical practice. Evidence based Clinical Practice Guidelines are being used increasingly as a strategy for achieving consistency across clinical areas and within practice. While there is increasing evidence of patient benefit from the utilisation of Clinical Practice Guidelines, clinician adherence and implementation remains poor and the failure to change clinician behaviour with the implementation of guidelines has been demonstrated in many areas of healthcare [1-3].

One such example of poor compliance with guidelines relates to the prevention of venous thromboembolism (VTE) for hospitalised patients. VTE manifests as deep vein thrombosis (DVT) and/or pulmonary embolism (PE). It spans hospital specialties and is usually incidental to the problem with which a patient presents to hospital. Nonetheless, the consequences of VTE are substantial. VTE contributes to as many as 10% of hospital deaths [4-6]. In addition, it is associated with significant morbidity from recurrent thrombotic events, post thrombotic syndrome (characterised by debilitating leg pain, swelling and fibrosis, and in severe cases, leg ulcers [7]) and pulmonary hypertension [8]. As such VTE results in major socioeconomic decrements in terms of family disruption and loss of time in the workforce for these patients. In Australia annual VTE associated costs, including the costs associated with premature death, are said to be as much as 0.15% of the Gross Domestic Product [9]. Effective prevention of VTE will save lives, improve patient outcomes and reduce healthcare costs.

Prevention of VTE for hospitalised patients is relatively easy and inexpensive. Evidence based guidelines (e.g. [10,11]) and Government policies (e.g. [12]) advocate risk stratification screening of patients and the use of thromboprophylactic medications (daily or twice daily sub-cutaneous injections of anticoagulants) or mechanical devices (e.g. wearing graduated compression stockings) for high risk patients for the period of their hospitalisation. However, large international audits have shown that thromboprophylaxis is not consistently applied by clinicians in hospital practice: only 39% of 'high-risk' medically ill patients and 58% of 'high-risk' surgical patients were receiving appropriate prophylaxis [6,13,14].

A number of strategies have been tried to improve risk assessment and thromboprophylaxis implementation. Like implementation strategies for numerous other guidelines [3], the strategies used for VTE prophylaxis guideline implementation have included education programs, computer and human reminder programs, audits and feedback. However, like other guideline implementation studies none have been overwhelmingly successful in achieving clinician behaviour change, and even the most successful usually fail to achieve sustainable change over time [15].

Grimshaw *et al. *[3] recommend that effective guideline implementation requires exploration of clinician practice and behaviour. Therefore, the aim of this study was to i) identify doctors' attitudes towards VTE prophylaxis; ii) understand the clinical environment in which VTE prophylaxis is implemented, and thus try to elucidate the barriers to clinicians implementing VTE Clinical Practice Guidelines and obtain stakeholder input and ideas so that appropriate strategies for practice change can be developed.

## Methods

Exploratory descriptive study methods [16] using face to face semi-structured interviews were implemented to understand attitudes and the clinical environment surrounding the application of VTE Clinical Practice Guidelines for hospitalised patients.

### Study setting and population

The study sites chosen were two tertiary referral hospitals in Sydney, Australia. Both sites are located within one area health service and are metropolitan based. Data was collected between February 2009 and August 2009 at the first hospital (n = 34 interviews), and in August 2009 at the second (n = 6 interviews). The interviews undertaken at the second hospital were intended to confirm or counter findings from the first site and thus to clarify whether the findings may have been institution specific. At the time of the study both hospitals had policies in place recommending VTE and bleeding risk assessment of all patients admitted. Both hospital policies list potential VTE risk factors and as per the recommendations of the Australian and New Zealand (ANZ) guidelines that were current at the time [17] recommend that in the presence of any VTE risk factor that VTE prophylaxis be implemented: chemical prophylaxis in the absence of bleeding risk factors and mechanical prophylaxis in all other patients. Both hospitals have haematologists with particular research interests in VTE and the main hospital has a history of VTE education sessions at Grand Rounds and a year prior to the current study had participated in a study with multiple education interventions intended to improve VTE prophylaxis [18].

A convenience sample of doctors working within both hospital sites at the time of the study were eligible to enrol. Representation was sought from a variety of medical and surgical specialties although Paediatrics, Radiology, Dermatology and Mental Health clinicians were excluded from the study due to there being few patients at high risk of VTE and thus few who need prophylaxis in these therapeutic areas.

Junior doctors (Interns and Registrars (JMO)) were enrolled at different stages within their medical training. They were randomly selected using the hospital database paging system. Those who answered their page were provided with a study description and invited to take part at a location and time of their convenience. Senior doctors (both physicians and surgeons) recruited for the study and were initially invited by email. Recruitment continued until new interviews were generating no new themes i.e. data saturation was achieved.

### Interview tool

Fourteen semi-structured open-ended questions (in a format recommended by Creswell and Clark [19]) were designed to explore clinicians' opinions, knowledge, beliefs and usage of Clinical Practice Guidelines. The questionnaire (see table 1) was designed to be non-leading with deliberate exclusion of key words. All interviews were conducted by a researcher independent of the hospital and its staff. Questions were conveyed in a casual conversational manner in order to establish rapport. This approach allowed respondents to express their opinions and ideas freely without bias or pre-conceived ideas imposed by the investigators.

**Table 1:** Questions used to direct discussion with doctors to collect information about their attitudes to VTE prophylaxis and the barriers and facilitators to them adopting VTE Clinical Practice Guidelines

1	When you are presented with a patient with a particular problem how do you ensure the appropriate care is provided and no aspect of care is overlooked?
2	How are changes or new research findings incorporated into your practice?

3	Some people think there is some variability in the practice of medicine. Why do you think this perception exists? How is consistency of treatment ensured in your area?

4	In general what do you know and how do you feel about VTE prophylaxis?

5	What influences whether a patient is VTE risk assessed in your ward and who oversees the assessment? What is the process in different parts of the hospital for a patient receiving or not receiving VTE prophylaxis? What is your role?

6	How often do you check junior staff are doing what you assume should be done and what you have asked to be done?

7	Can you tell me about a particular patient on your ward that is now receiving
	i. Anticoagulant prophylaxis?
	ii. Mechanical prophylaxis?
	iii. Not receiving either?

8	What was it about that patient or their management that has resulted in them receiving or not receiving prophylaxis? Would you manage medical patients differently to surgical patients with regards to VTE prophylaxis?

9	How have you gained your information and knowledge about VTE prophylaxis?

10	Do you know of anyone who has had poor outcomes because of VTE prophylaxis, or lack thereof? Has it changed your perspective?

11	How do you feel about the way this hospital manages/uses VTE prophylaxis in general?

12	In an ideal world where resources are not an issue how would you ensure VTE prophylaxis is optimized at this hospital? *

13	I will now give you the official guidelines that are endorsed by this hospital, please take a moment to review them. Are you familiar with them? *

14	Do you have any thoughts on them, on their improvement or otherwise. *

* Questions added after completion of first 8 interviews.

### Ethics

The study was approved by both hospitals' Human Research Ethics Committees as well as that of the University of New South Wales. All participants gave written informed consent for their interview to be recorded and analysed. All data was de-identified and remained anonymous for analysis. Data was stored and maintained within a password protected file.

### Data Collection

The interviews were recorded using an Olympus digital voice recorder (DS-3300) and transcribed verbatim into Microsoft Word and saved as 'read only' files for analysis. Demographic data (including age, current ward, years of experience, and time working in the current role) was obtained from each participant at the time of interview.

### Data analysis

Interview data was transcribed by the researcher who conducted the interviews. Interview transcripts were then analysed using coding methods described by Creswell and Clark [19]. This included line-by-line open coding where conceptual labels within the transcripts were developed and then grouped into categories. A qualitative software program (NVivo™) assisted managing data files by linking codes and categories, and organising ideas into main themes. Initial coding of the data in six transcripts was conducted independently by three co-investigators. The coding was then reviewed, rationales for codes were discussed, and some categories were changed as a result. After the intra-rater checks were completed, a single coder completed coding and analysis of the remaining data.

## Results

**Figure 1:**
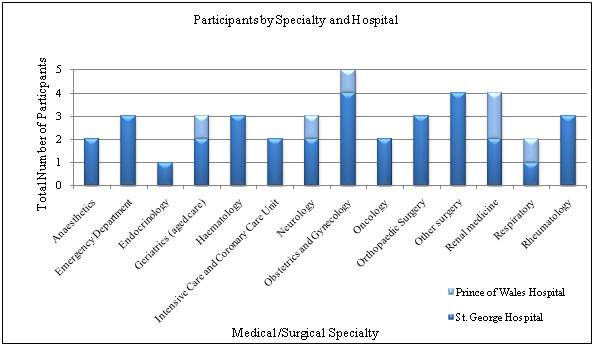
**Data displaying medical and surgical specialties of clinicians interviewed (n = 40)**.

Forty clinicians from medical and surgical specialties, and different hierarchical role positions took part in the study. Details of medical specialities enrolled are represented in Figure 1. The interview participants' demographic data are presented in Table 2. Complete interviews were conducted with 39 of the 40 interviewees. One interview was incomplete as the participant was called to a medical emergency and did not wish to resume the interview. The average interview duration was 20 minutes. There were no thematic differences identified in the data collected from the two hospital sites.

**Table 2:** Demographic data for participants (n = 40)

Characteristic	Value
Gender	
Female, n (%)	23 (58%)

Age, mean (SD)	33.3 (11.1)

Hospital, n (%)	
St. George Hospital	34 (85%)
Prince of Wales Hospital	6 (15%)

Clinical Specialty, n (%)	
Medical	26 (65%)
Surgical	14 (35%)

Position, n (%)	
Senior Clinician	12 (30%)
Registrar	8 (20%)
Resident Medical Officer/Intern	20 (50%)

Years in current position, mean (SD)	5.4 (7.6)

Abbreviations: SD, Standard Deviation

Interviews identified three key themes influencing clinician behaviour and beliefs with respect to VTE Clinical Practice Guidelines: 1) 'Guidelines: friends or foe'-clinicians' attitudes about VTE thromboprophylaxis and guidelines; 2) 'Practice Culture' which describes an environment in which medical team rejection, adherence or utilisation of guidelines is driven by team member influence; and 3) 'Fragmentation of Care'-a major barrier to guideline compliance, VTE risk assessment screening, and implementation of medication prophylaxis in hospitalised patients.

### Guidelines Friends or Foe: Attitudes towards thromboprophylaxis and the VTE Clinical Practice Guidelines

Under this theme we describe an attitude which, despite general support for the VTE prevention guidelines, does not encourage the changes in clinical behaviour patterns necessary to adhere to guidelines.

There was a 100% consensus among interviewees that VTE is a risk hospitalised patients are exposed to, and that it is worth preventing:

*DVT is a common thing, potentially life threatening and for patient care it's a good idea [to prevent it]*. Interview 43-Junior Medical Officer, Medical ward

There was agreement by participants that current implementation of VTE prophylaxis medication was either "good" or "appropriate" in their institutions and most (62%) were unaware of audit data (locally or internationally) showing the implementation of prophylaxis in hospitalised patients is often suboptimal or inappropriate. All interviewees felt that thromboprophylaxis risk assessment screening and anticoagulant prescribing was a doctor's responsibility and the majority (86%) had some awareness of the existence of Hospital Clinical Practice Guidelines for VTE prophylaxis. However, only a few clinicians reported using the guidelines when they reviewed patients:

*I actually don't use [guidelines]. I just write up what I know which is Heparin 5000 BD or TDS... I don't know what the guidelines say*. Interview 4-Junior Medical Officer, Medical specialty

*It's a willy nilly approach to each patient. So its "Oh, this patient is a bit overweight, yeah let's [prescribe] them [prophylaxis]". Or someone else might say, "Oh well, they're a bit overweight, but it's not too bad" and won't [prescribe] them [prophylaxis]. You sort of learn haphazardly who to [treat]. You don't refer [to] protocols to do it*. Interview 34-Registrar, Medical specialty

Participants described how senior clinicians practice an 'art of medicine' which has been defined elsewhere as the combination of medical knowledge, intuition, experience and judgement [20]. Clinicians develop preferences or an 'art' towards patient management and treatment medicine which is considered to take precedence over guidelines. With respect to VTE prophylaxis, clinicians reported taking guidelines into consideration but rarely considered they should or could be applied universally.

*Medicine is not a science, it's an art, so you cannot implement a protocol to every single patient*. Interview 14-Registrar, Surgical ward.

Thus senior clinician's behaviour often intentionally deviated from guideline practice. Based on their personal experiences they reported making conscious decisions not to implement evidence based guidelines. Senior clinicians reportedly were motivated to consider either applying prophylaxis or withholding prophylaxis because they wished to prevent litigation or retribution, or to prevent a past adverse experience when one of their patients was or was not given VTE prophylaxis.

*If anything when VTE prophylaxis is considered, it's prescribed in excess of these [Australasian National] guidelines; a lot of people who are low risk are still given VTE prophylaxis. I think it's just defensive medicine, 'cause I guess in our minds a person who is low risk is also not likely to bleed so it's safer just to give them VTE prophylaxis 'cause then you could never be accused of not giving VTE prophylaxis if they had a PE or something*. Interview 1- Junior Medical Officer, Medical ward

*You will find that if a consultant has had a particularly severe adverse event from something, they will be reluctant to use it whereas it might be evidence based best practice to do so*.

Interview 39-Junior Medical Officer, Medical ward

Senior clinicians who had had a patient who suffered or died from a pulmonary embolism as a result of a lack of prophylaxis were more likely to be conscientious with subsequent patients.

*If you're working on the surgical ward and you see someone get PE and have a bad outcome, you're much more vigilant in the future about trying to prevent it*.

Interview 43-Junior Medical Officer, Surgical ward

However, a significant number of interviewees seemed to be overly concerned with their patients being at risk of bleeding as a result of prophylaxis with anticoagulant medication (on occasion following experiencing having a patient with a serious anticoagulant related bleed). They used this concern as justification for not using anticoagulant medication especially when combined with their strong desire to do 'no harm' to their patients.

*Some people use a lot more prophylaxis than I would. I just think that, well I think that you can cause harm with it so you should use it evidence based for a start, it's got to be high risk, you can cause harm with [prophylaxis]*. Interview 10-Registrar, Surgical ward

*I think in the physician group its oversight [of VTE prophylaxis] and in the surgical group its paranoia. I think in many cases the fear [of bleeding] is overstated particularly in neurosurgery and even more so in orthopaedics*. Interview 23-Senior Clinician, Medical ward

Contrary to the Australian guideline recommendations [17] that were in use within both of our institutions, the majority of interviewees reported that their primary criteria for deciding whether a patient should receive prophylaxis medication or not, was the patient's mobility or immobility.

*I think immobility is probably the biggest [indication]. [Patients who] are immobile and have no other contraindications [will get prophylaxis]. The ones who are walking [around] might not receive any [prophylaxis]*. Interview 14-Registrar, Medical specialty

*I think DVT Prophylaxis in some form should be compulsory for every single patient that presents in to hospital, even mobile patients. I think that in hospital people are still much less mobile than when they are at home or in their work setting. So even if someone's mobile the reality is they are just probably mobilising to the toilet and back*.

Interview 40-Registrar, Medical specialty

Thus although the majority of interviewees had no particular concerns with the VTE Prophylaxis Clinical Practice Guidelines, the prevailing medical behaviour adhered to 'adapting practice' for individual patients rather than the 'cook-book' approach of the Clinical Practice Guidelines.

### Culture of Practice: Drivers of whether evidence based best practice is implemented

Organisational culture is seen as a set of norms, rules, values, philosophies, attitudes, and meanings distributed within a group and that manifest in their behaviour and thus their observable practices [21]. The 'Culture of Practice' described in our interviews was one of a very hierarchical team environment where senior clinicians prescribe practices within their teams. Our hospitals as organisations are an environment in which we saw the 'community of practice' described by Fenlie *et al. *[22] where one doctor within a team cannot adopt significant changes to practice without discussion and consent from colleagues within the team. VTE prophylaxis practice varied from one ward to another and from one senior clinician to another. Junior staff reported adapting their behaviour and practices to that of their current senior clinician and medical team.

*The biggest influence [on my practice] is just what my seniors do on that term, so my practice would change from term-to-term depending on what my seniors do and want*.

Interview 26-Junior Medical Officer, Surgical Ward

*Yeah, I've looked at [the VTE prevention guidelines] a couple of times, mostly though you just get into a pattern of what your bosses like and that's what you end up doing*.

Interview 26-Junior Medical Officer, Surgical ward

However with no consistency of practice and constrained by the need to conform with team behaviour and actions, Junior Medical Officers (JMOs) often lack the confidence required to apply evidence based practice.

Thus interviewees identified a team approach that was focused on shared values and beliefs about practice, which were largely directed by senior clinicians' preferences often despite prevailing evidence. Junior Medical Officers embraced the team culture modifying their own behaviour to align it with that of more senior team members.

### Fragmentation of Care: A barrier to evidence based best practice

'Fragmentation of care' is used to describe specialty based medical care which has led to some responsibilities being unclear. Compounding the situation is the often less than ideal communication between the hospital team members themselves. Interviewees described VTE prophylaxis 'falling through the gaps' due to oversight. It was overlooked as focus was being given to the presenting illness and treating the medical symptoms rather than problems that may or may not develop throughout the hospitalisation.

*Well it never is the first priority preventing DVT/PE. I mean the first priority is to stabilise the patient and [manage their] condition and [VTE prophylaxis] doesn't come under there, but it should come under your overall management plan of the patient but it gets overlooked I think... it's easy to overlook it*. Interview 11-Junior Medical Officer, Medical specialty

*You've got a patient who has come in with a problem or a symptom and you're concentrating on that and if that isn't the thromboembolic family, then you're not necessarily going to think about [VTE]*. Interview 47-Senior Clinician, Emergency Medicine

'Specialisation' of care was also used to explain VTE 'blindness' by staff: clinicians' practice is focused on providing medical treatment for particular areas of specialty rather than holistic patient care. Specialisation contributes towards the fragmentation of care.

*You know they are too focused on the heart or the kidney or whatever it is they are doing to think about DVT prophylaxis*. Interview 23-Senior Clinician, Medical ward

Generally, there was a lack of consistency about whose responsibility it was to consider VTE screening and prophylaxis medications. This was in terms of which medical team speciality was responsible for this intervention, as well as which member of the medical team should undertake the screening and write up the medication order (junior or senior medical staff). Although, there was consensus that VTE risk assessment should occur early in a patient's admission, different medical specialties disputed whose role it was to consider VTE screening and prophylaxis medications. For example, there was some dispute amongst participants as to whether VTE screening should be conducted by Emergency staff or by the admitting medical in-patient team.

*There is a bit of confusion in [the Emergency Department] about whose job it is to chart the patient's med[ication]s, whether it's the doctor that sees them in [the Emergency Department] or the admitting registrar or the team on the ward*. Interview 26-Junior Medical Officer, Surgical ward

*I don't think [VTE prophylaxis] necessarily fits into my initial responsibility of care. If I'm admitting a patient directly under the [Senior Clinician], then it probably does fit under my guides of care, I think it should be a shared responsibility, not entirely our responsibility to [chart prophylaxis]. [And] it's just a matter of priorities, I think in [the Emergency Department] 'cause we are always under time [pressure]*. Interview 20-Senior Clinician, Emergency Medicine

Role confusion with respect to thromboprophylaxis was even more evident when multiple medical teams were caring for patients, especially when the patient had been admitted to hospital with multiple co-morbidities. The following clinicians recount how assumptions with regards to thromboprophylaxis may lead to patients 'falling through the gaps':

*Unfortunately what happens sometimes is a patient gets admitted under one [Senior Clinician], gets operated [on] by another; a different registrar comes on to take care of them on the ward-it's like someone will deal with it, not me*. Interview 34-Registrar, Surgical ward

*Well one reason is if you're the second person or third person to see the patient you might assume that [VTE prophylaxis] is already there*. Interview 11-Junior Medical Officer, Medical ward

After-hours care puts patients at greater risk of being missed for VTE prophylaxis as hospital staffing numbers and experience levels are commonly reduced. Participants identified that lower after-hours staffing and expertise reduced opportunities to consider VTE risk and that patients coming into hospital, after-hours, would often fall through the gaps.

*On Monday after the weekend intake, some of the teams will have 30 or 40 patients to see and they won't always get to [review and chart VTE prophylaxis]*. Interview 27-Junior Medical Officer, Surgical ward

*I think that variability often happens on weekends or after-hours. [Those] doctors don't always know the story and it's hard when you've got lots of patients to see and to prioritise... to be honest [after hours] you'd just see patients who are [really] sick, so I don't always check if they are on prophylaxis, I don't have the time*. Interview 12-Junior Medical Officer, Surgical Ward

Greater than 25% of participants interviewed who were part of a medical ward admitting team volunteered that if VTE prophylaxis was omitted during the initial phases of a patient's admission that the omission could remain unnoticed for several days, or in some cases, for the remainder of the patient's hospital stay:

*From my experience it's great if they're flagged and charted for prophylaxis at admission. If not, they tend to sort of get left for a few days before you realise. You sort of assume it has been addressed and it's only later you find out that it hasn't [been]*. Interview 13-Junior Medical Officer, Medical ward

*If it's not remembered at admission then no one remembers it a week down the track and then its two weeks down the track and your patient has a PE, so I think that's one problem*.

Interview 1-Junior Medical Officer, Medical ward

In addition to a lack of clarity between medical teams, there was no clarity about who is responsible within a team for VTE risk assessment. Although, with the exception of one, all senior clinicians interviewed felt that VTE prophylaxis management could be delegated to junior staff members.

*Mostly in my ward [VTE prophylaxis is] not my duty but the duty of the junior... medical officers*. Interview 2-Senior Clinician, Medical ward

However, it appeared that junior doctors infrequently initiate VTE prophylaxis without direction.

*It was my first term and I felt like I was too junior to be deciding what-I wasn't confident in prescribing VTE prophylaxis*. Interview 26-Junior Medical Officer, Surgical ward

*I'm not sure that as a new intern [they will] put someone on prophylaxis unless the registrars tell them to*. Interview 14-Registrar, Surgical ward

Thus in a time poor environment with multiple teams and multiple clinicians within the teams contributing to the care of an individual patient, the cross functional responsibility of VTE implementation and management is susceptible to being overlooked.

### Strategies suggested to improve evidence based practice

Acknowledging that VTE is not a priority, the interviewees repeatedly highlighted how time poor they were when reviewing individual patients. Thus for VTE risk assessment screening to be incorporated into everyday practice, it needs to be easy to implement, and implemented in some sort of routine way. Interviewees recommended that there should be simple reminders that could be used as a record of risk assessments having been completed.

[Risk assessment] should just be part of the standard chart like the allergy form is...

Interview 1-Junior Medical Officer, Medical ward

*It's probably best to assign one person... someone at the ward level who checks it for everyone*.

Interview 2-Senior Clinician, Medical ward

*I guess it depends on making it a habit*. Interview 3-Junior Medical Officer, Medical ward

*I think [it should be] in the chart... like a DVT prophylaxis little box and that way you could also mark, "no DVT prophylaxis" and a bit [of information on] why [it's omitted] so that if the situation changes or any new teams come into the picture they know why you've chosen not to [prescribe]*. Interview 13-Junior Medical Officer, Medical ward

*Ideally you would have a DVT clinical nurse consultant who went around and looked at every med chart. I think a human reminder is still the best*. Interview 16-Senior Clinician, Medical ward

*I think the opt-out system is the best and in an ideal world there would be some type of automatic alert system whereby if a patient is admitted to the ward a certain number of things have to be checked off on a computer as part of that admission*. Interview 33-Senior Clinician, Medical ward

Interviewees suggested a sticker in the medical charts may be an appropriate prompt for staff to undertake VTE screening. Interestingly, our primary study site uses a prompting sticker system. A recent medical record audit we conducted identified that all patients had the bright blue prompt sticker in the notes. However, few if any of the stickers had been completed.

## Discussion

Guidelines are intended to 'improve quality of care by decreasing inappropriate variation and expediting the application of effective advances to everyday practice'[2]. Adoption of VTE prevention guidelines is expected to save lives, improve patient outcomes and reduce healthcare costs. Nonetheless, like many areas of healthcare [1,23], adoption of VTE prevention guidelines is suboptimal [13,14]. This study gathers information about clinician attitudes and the clinical environment in which clinicians are working and establishes that the barriers to implementing Clinical Practice Guidelines for VTE prophylaxis are multi-factorial. It is not surprising therefore that single approaches to improving VTE prevention are rarely effective [15]. However as recommended in practice change literature [22,24-28], having identified the barriers, multilevel and systematic strategies for changing practice can be proposed.

The attitudes towards VTE prophylaxis and the guidelines that were elucidated in the interviews suggest that in the first instance individual clinicians need to be convinced that change is necessary. Clinicians need to know how their practice differs from guideline recommendations and then they need to believe that changing to guideline recommendations will improve outcomes for their patients. The majority of our interviewees believed that VTE prophylaxis was well implemented in their hospital. This is contrary to what is observed internationally [13,14], but also contrary to audit data collected coincidentally at the primary study site (unpublished data) where we found 36% of patients at high risk for VTE are overlooked for prophylaxis during their hospitalisation. A traditional audit and feedback approach could well address this barrier to guideline adoption.

More difficult to address is the need to convince clinicians that following the guideline recommendations will be in their patient's best interests. The current environment in Australia promotes the use of VTE prophylaxis in hospitalised patients: the National Health and Medical Research Council has identified VTE prevention as a priority area for improving patient safety and has generated national guidelines [11] and State Health Departments are preparing policies to enforce risk assessment and VTE prophylaxis implementation. The result of this activity is perhaps the heightened awareness evident in the interview data of VTE as a condition of importance and a condition worth preventing. However although, all our interviewees knew guidelines existed, and 98% expressed an opinion consistent with support for VTE prevention guidelines, clinicians, particularly those managing medical patients, considered that the guidelines were not intended to be applied universally but more to act as a guide for adapting practice to individual patients ('the art of medicine'). We found personal experiences rather than guidelines dominate practice behaviour, beliefs and values. Instead of referring to guidelines to guide clinical decision making and practice, each clinician's practice is based on their experiential knowledge gained and continually updated and amended during their education, conferences, discussions with colleagues and management of individual patients. Gabbay and LeMay [29] describe this acquisition of knowledge for applying to medical practice as developing 'mindlines' rather than guidelines. This was evident by interviewees exaggerating the bleeding risks associated with anticoagulants at prophylactic doses and exaggerating immobility as a VTE risk factor, and by the variations in team practice described by junior doctors. If guideline directed care for VTE prophylaxis is going to be embraced by clinicians, there has to be clear evidence that its implementation will add benefit to their current practice for patients.

Perhaps it is time to alter traditional approaches to rolling out Clinical Practice Guidelines. Like the majority of evidence based guidelines, guidelines for VTE risk assessment and prophylaxis application are based on clinical trial evidence. However, clinical trials by their very nature include only a select group of patients [30,31], and guidelines are an extrapolation from these select groups to broader population groups. For example Tapson et al. [32] report that less than 12% of real world medical patients fulfil VTE prophylaxis clinical trial inclusion criteria. Although, VTE prevention guidelines are generally accepted and advocated [10,33,34] within the medical community, they are not completely without controversy because of the clinical trials upon which they are based [35,36]. If a research study demonstrated that consistent application of VTE guidelines results in an improvement in clinical outcomes (in this case less deep vein thrombosis, pulmonary embolism, and bleeding events) as well as being cost effective, would senior clinicians be sufficiently motivated to switch to a 'cookbook' approach? It is an approach to rolling out treatment guidelines that has yet to be tried at least in VTE prophylaxis.

Sustainable changes to clinical practice need to be driven and championed by individuals. As our exploration into the clinical environment suggested, to encourage a culture of evidence based practice within health care systems, senior clinician support is critical. The participants in our study describe a culture where there is already very little variation within a team (the senior clinician leading the team prescribes the practice of the clinicians within the team). The social context of practice is autonomous and hierarchical with junior staff altering their behaviour to reflect that of their current team's senior clinician. It is one which fosters a team approach and fosters consistency within that team. In this way organisational culture can be remoulded. In the case of VTE prophylaxis, our results suggest that if senior clinicians' practice is consistent with guidelines, members of their teams will follow suit.

However the clinical environment we described was also one of competing priorities. VTE risk crosses all medical specialties and as such needs to be considered for nearly every patient admitted to hospital. Even with the best intentions, with the multitudes of competing priorities when a patient is first admitted to hospital, and with the inherent care fragmentation of multiple teams and multiple members of teams looking after each patient, it is regularly overlooked. Role confusion within teams, and between teams, was a recurring theme and is consistent with the findings of Cook *et al. *[37] who postulate that the intra- and multidisciplinary care inherent in hospital patient management, results in unclear role accountability. The World Health Organisation listed communication during patient care handover as one of its 'high 5' patient safety initiatives to improve continuity, safety and ultimately the outcome of care [38]. Interviewees reported that communication between teams about whether risk assessment had been done was lacking. The lack of adequate documentation adds to role responsibility confusion- who in the team as well as which team should be managing VTE prophylaxis?

Role responsibility needs to be clarified across the organisation and systems need to be implemented to facilitate documentation and handover communication in the fragmented system of care functioning in hospitals today. VTE prophylaxis is more consistently applied to surgical patients than medically ill patients [14]. Since the incidence of VTE in untreated patients varies little between medical and surgical patients [10], the difference may be due to the fact that the importance of VTE prophylaxis for surgical conditions has been supported for a much longer period of time. It may be related to the fact that VTE guidelines are more consistent and easier to apply in the surgical patient cohort. Alternatively, it may be related to its routine consideration and charting as part of the pre-operation work up for the patient. By having guidelines embedded in structural processes and clearly articulating role responsibilities with regards to VTE risk assessment and prophylaxis implementation, it seems it is more likely to be considered and appropriately prescribed.

The fact that clinicians do not routinely refer to guidelines, that they are time poor and have many competing priorities, suggests that tools to address the fragmented care present must be simple and effective. These could take the form of reminders (e.g. computer alerts or human reminders or less sophisticated paper cues (e.g. lists and stickers)). Kucher *et al *[39] have demonstrated that a computer alert on electronic medical records decreased the occurrence of DVT and PE by 41% compared to patients whose doctors were not prompted for VTE prophylaxis with an electronic-reminder [39,40]. In the absence of suitable technology, having a dedicated VTE prophylaxis support position such as a nurse practitioner within a hospital has been demonstrated to improve prophylaxis rates by up to 48% [18,40].

Thus the attitudes of doctors to VTE prevention and evidence based practice with respect to VTE prevention, and clinical environment in which VTE prevention is implemented, suggest that like the change theories of Grol and Grimshaw [27], changing current practice with respect to VTE prevention will need to incorporate a multilevel approach taking into consideration the individual doctors, the social context in which they work as well as the organisational context.

## Conclusion

The complexity and drivers of clinical behaviour and decision making provides a significant challenge to the implementation of evidence based practice guidelines in our health system. However patient outcomes can be enhanced through the utilisation of evidence based practice guidelines and the best possible approach to improving patient outcomes associated with VTE is a coordinated program that addresses individual, cultural and organisational constraints.

## Competing interests

The authors declare that they have no competing interests.

## Authors' contributions

NHC participated in study design and coordination, and data analysis, and drafted the manuscript. SPL conducted the interviews, participated in the data analysis and helped to draft the manuscript. MF participated in the data analysis and helped to draft the manuscript. MNL conceived the study and participated in the design and coordination and helped draft the manuscript. BHC conceived the study and participated in the design and coordination and helped draft the manuscript. All authors read and approved the final manuscript.

## Pre-publication history

The pre-publication history for this paper can be accessed here:

http://www.biomedcentral.com/1472-6963/11/240/prepub

## References

[B1] Grol R Successes and failures in the implementation of evidence-based guidelines for clinical practice Med Care 2001 39 8 Suppl 2 II46 54 11583121 10.1097/00005650-200108002-00003

[B2] Cabana MD Rand CS Powe NR Wu AW Wilson MH Abboud PA Rubin HR Why don't physicians follow clinical practice guidelines? A framework for improvement JAMA 1999 282 15 1458 1465 10.1001/jama.282.15.1458 10535437

[B3] Grimshaw JM Thomas RE MacLennan G Fraser C Ramsay CR Vale L Whitty P Eccles MP Matowe L Shirran L Wensing M Dijkstra R Donaldson C Effectiveness and efficiency of guideline dissemination and implementation strategies Health Technol Assess 2004 8 6 iii iv 1-72 14960256 10.3310/hta8060

[B4] Anderson FA Jr Wheeler HB Goldberg RJ Hosmer DW Forcier A Patwardhan NA Physician practices in the prevention of venous thromboembolism Ann Intern Med 1991 115 8 591 595 1892330 10.7326/0003-4819-591

[B5] Sandler DA Martin JF Autopsy proven pulmonary embolism in hospital patients: are we detecting enough deep vein thrombosis? J R Soc Med 1989 82 4 203 205 2716016 10.1177/014107688908200407 PMC1292084

[B6] Cohen AT Agnelli G Anderson FA Arcelus JI Bergqvist D Brecht JG Greer IA Heit JA Hutchinson JL Kakkar AK Mottier D Oger E Samama MM Spannagl M Venous thromboembolism (VTE) in Europe. The number of VTE events and associated morbidity and mortality Thromb Haemost 2007 98 4 756 764 17938798 10.1160/TH07-03-0212

[B7] Prandoni P Lensing AW Cogo A Cuppini S Villalta S Carta M Cattelan AM Polistena P Bernardi E Prins MH The long-term clinical course of acute deep venous thrombosis Ann Intern Med 1996 125 1 1 7 8644983 10.7326/0003-4819-125-1-199607010-00001

[B8] Pengo V Lensing AW Prins MH Marchiori A Davidson BL Tiozzo F Albanese P Biasiolo A Pegoraro C Iliceto S Prandoni P Thromboembolic Pulmonary Hypertension Study Group Incidence of chronic thromboembolic pulmonary hypertension after pulmonary embolism N Engl J Med 2004 350 22 2257 2264 10.1056/NEJMoa032274 15163775

[B9] Access-Economics The burden of venous thromboembolism in Australia. Report for the Australian and New Zealand Working Party on the Management and Prevention of Venous Thromboembolism 2008 http://www.accesseconomics.com.au

[B10] Geerts WH Bergqvist D Pineo GF Heit JA Samama CM Lassen MR Colwell CW Prevention of venous thromboembolism: American College of Chest Physicians Evidence-Based Clinical Practice Guidelines (8th Edition) Chest 2008 133 6 Suppl 381S 453S 10.1378/chest.08-0656 18574271

[B11] National-Health-and-Medical-Research-Council-Commonwealth-Government-of-Australia Clinical Practice Guideline for Preventing Venous Thromboembolism in Patients Admitted to Hospital 2009

[B12] Committee HoCh The Prevention of Venous Thromboembolism in Hospitalised Patients Second Report of Session 2004-2005 2005 Published on 8 March 2005 by authority of the House of Commons London: the Stationery Office Limited

[B13] Tapson VF Decousus H Pini M Chong BH Froehlich JB Monreal M Spyropoulos AC Merli GJ Zotz RB Bergmann JF Pavanello R Turpie AG Nakamura M Piovella F Kakkar AK Spencer FA Fitzgerald G Anderson FA Jr for the IMPROVE Investigators Venous thromboembolism prophylaxis in acutely ill hospitalized medical patients: findings from the International Medical Prevention Registry on Venous Thromboembolism Chest 2007 132 3 936 945 10.1378/chest.06-2993 17573514

[B14] Cohen AT Tapson VF Bergmann JF Goldhaber SZ Kakkar AK Deslandes B Huang W Zayaruzny M Emery L Anderson FA Jr Venous thromboembolism risk and prophylaxis in the acute hospital care setting (ENDORSE study): a multinational cross-sectional study Lancet 2008 371 9610 387 394 10.1016/S0140-6736(08)60202-0 18242412

[B15] Sliwka D Fang MC Venous thromboembolism prophylaxis in the United States: still room for improvement J Gen Intern Med 2010 25 6 484 486 10.1007/s11606-010-1350-9 20390463 PMC2869419

[B16] Stewart A The ethnographer's method. Qualitative Research Methods 1998 46 Sage Publications, California

[B17] Fletcher JBR MacLellan D Chong BH Fisher C Gallus A Gibbs H Hannan T Matthews G Salam H Stacey M van Rij A Prevention of venous thromboembolism: Best Practice Guidelines for Australia and New Zealand Health Education and Management International, Sydney 2009 4

[B18] Gibbs H Fletcher J Blombery P Glennane A Collins R Does a dedicated nurse practitioner improve thromboprophylaxis use in acutely ill medical patients in Australia? The methodology for a multicentre VTE Task Force Audit Int Angiol 2009 28 1 73 78 19190560

[B19] Creswell JQPCV Designing and Conducting Mixed Methods Research 2007 Sage Publications, California

[B20] Fauci ABE Kasper D Harrison's principles of Internal Medicine 17th Edition 2008 McGraw-Hill Companies

[B21] Nugus P Braithwaite J The dynamic interaction of quality and efficiency in the emergency department: Squaring the circle? Soc Sci Med 2010 70 4 511 517 10.1016/j.socscimed.2009.11.001 19942332

[B22] Fenlie EFL Wood M Hawkins C The nonspread of innovations: the mediating role of professionals Academy of Management Journal 2005 48 1 117 134

[B23] Oxman AD Thomson MA Davis DA Haynes RB No magic bullets: a systematic review of 102 trials of interventions to improve professional practice CMAJ 1995 153 10 1423 1431 7585368 PMC1487455

[B24] Craig P Dieppe P Macintyre S Michie S Nazareth I Petticrew M Developing and evaluating complex interventions: the new Medical Research Council guidance BMJ 2008 337 a1655 10.1136/bmj.a1655 18824488 PMC2769032

[B25] Grol R Personal paper. Beliefs and evidence in changing clinical practice BMJ 1997 315 7105 418 421 9277610 10.1136/bmj.315.7105.418 PMC2127297

[B26] Campbell NC Murray E Darbyshire J Emery J Farmer A Griffiths F Guthrie B Lester H Wilson P Kinmonth AL Designing and evaluating complex interventions to improve health care BMJ 2007 334 7591 455 459 10.1136/bmj.39108.379965.BE 17332585 PMC1808182

[B27] Grol R Grimshaw J From best evidence to best practice: effective implementation of change in patients' care Lancet 2003 362 9391 1225 1230 10.1016/S0140-6736(03)14546-1 14568747

[B28] Greenhalgh T Robert G Macfarlane F Bate P Kyriakidou O Diffusion of innovations in service organizations: systematic review and recommendations Milbank Q 2004 82 4 581 629 10.1111/j.0887-378X.2004.00325.x 15595944 PMC2690184

[B29] Gabbay J le May A Evidence based guidelines or collectively constructed "mindlines?" Ethnographic study of knowledge management in primary care BMJ 2004 329 7473 1013 10.1136/bmj.329.7473.1013 15514347 PMC524553

[B30] Gross CP Mallory R Heiat A Krumholz HM Reporting the recruitment process in clinical trials: who are these patients and how did they get there? Ann Intern Med 2002 137 1 10 16 12093240 10.7326/0003-4819-137-1-200207020-00007

[B31] Tinetti ME Bogardus ST Jr Agostini JV Potential pitfalls of disease-specific guidelines for patients with multiple conditions N Engl J Med 2004 351 27 2870 2874 10.1056/NEJMsb042458 15625341

[B32] Tapson VFDH Decousus H Bergmann JF Chong BH Froehlich JB Kakkar AK Merli GJ Monreal M Nakamura M Pavanello R Pini R Piovella F Spyropoulos AC Turpie AGG Zotz RB FitzGerald G Anderson FA Jr for the IMPROVE investigators The International Medical Prevention Registry on Venous Thromboembolism (IMPROVE): Venous Thromboembolism Prophylaxis Practices in Acutely Ill Medical Patients Chest 2004 104 11 488a

[B33] Turpie AG Leizorovicz A Prevention of venous thromboembolism in medically ill patients: a clinical update Postgrad Med J 2006 82 974 806 809 10.1136/pgmj.2005.044107 17148703 PMC2653926

[B34] Dentali F Douketis JD Gianni M Lim W Crowther MA Meta-analysis: anticoagulant prophylaxis to prevent symptomatic venous thromboembolism in hospitalized medical patients Ann Intern Med 2007 146 4 278 288 17310052 10.7326/0003-4819-146-4-200702200-00007

[B35] Warwick D Samama MM The contrast between venographic and clinical endpoints in trials of thromboprophylaxis in hip replacement J Bone Joint Surg Br 2000 82 4 480 482 10.1302/0301-620X.82B4.9876 10855865

[B36] Segers AE Prins MH Lensing AW Buller HR Is contrast venography a valid surrogate outcome measure in venous thromboembolism prevention studies? J Thromb Haemost 2005 3 5 1099 1102 10.1111/j.1538-7836.2005.01317.x 15869620

[B37] Cook D Tkaczyk A Lutz K McMullin J Haynes RB Douketis J Thromboprophylaxis for hospitalized medical patients: a multicenter qualitative study J Hosp Med 2009 4 5 269 275 10.1002/jhm.461 19504488

[B38] Johnson JK Arora VM Improving clinical handovers: creating local solutions for a global problem Qual Saf Health Care 2009 18 4 244 245 10.1136/qshc.2009.032946 19651924

[B39] Kucher N Koo S Quiroz R Cooper JM Paterno MD Soukonnikov B Goldhaber SZ Electronic alerts to prevent venous thromboembolism among hospitalized patients N Engl J Med 2005 352 10 969 977 10.1056/NEJMoa041533 15758007

[B40] Huang A Barber N Northeast A Deep vein thrombosis prophylaxis protocol--needs active enforcement Ann R Coll Surg Engl 2000 82 1 69 70 10700773 PMC2503451

